# Supplementary material for: Observation of bound state self-interaction in a nano-eV atom collider
Source: Nat Commun. 2018 Nov 20;9:4895. doi: 10.1038/s41467-018-07375-8 (PMC6243998; doi:10.1038/s41467-018-07375-8)
Supplement: Supplementary file 1 — Supplementary Information [file 41467_2018_7375_MOESM1_ESM.pdf]

Supplementary Information for

Observation of bound state self-interaction in a nano-eV atom collider

R. Thomas, M. Chilcott, E. Tiesinga, A. B. Deb, and N. Kjærgaard

SUPPLEMENTARY NOTE 1

Derivation of scattered fraction

We use a simple analytical model to describe our measurements of the scattered fraction S as a function of the elastic scattering cross-section σ given in Eq. (5) of the main text. We start from the model used in Thomas *et al.*[1], which makes three assumptions. First, we replace the initial momentum distribution of the atoms by delta functions in momentum space corresponding to velocities $\mathbf{v}_1 = v_1\hat{\mathbf{z}}$ and $\mathbf{v}_2 = -v_2\hat{\mathbf{z}}$. Second, we ignore the dynamics of the scattered atoms, so we can represent scattered atoms as loss from the initial clouds. Third, we assume that the phase-space densities are sufficiently low to ignore quantum statistics. Taken together, these three assumptions reduce the quantum Boltzmann equation to a pair of coupled transport equations

$$(\partial_t + v_i\partial_z)n_i(\mathbf{r}, t) = -v\sigma n_1(\mathbf{r}, t)n_2(\mathbf{r}, t) \tag{1}$$

where the $n_i(\mathbf{r}, t)$ are the densities of the two clouds composed of either ^{40}K or ^{87}Rb, $v = |v_1 - v_2|$ is the magnitude of the relative velocity of the clouds, and σ is the cross section evaluated at the collision energy $E = (m_1v_1^2 + m_2v_2^2)/2$. We further assume that the densities remain Gaussian and can be written as

$$n_i(\mathbf{r}, t) = \frac{N_i(t)}{(2\pi)^{3/2}s_{x,i}s_{y,i}s_{z,i}}\exp\left(-\frac{x^2}{2s_{x,i}^2} - \frac{y^2}{2s_{y,i}^2} - \frac{(z - v_it)^2}{2s_{z,i}^2}\right), \tag{2}$$

where $N_i(t)$ is the time-dependent number of atoms and $s_{x,i}$ is the width of the cloud in the x-direction (and similarly for y and z). Supplementary Equation (1) can be integrated over x, y, and z to give

$$\frac{dN_i(t)}{dt} = -v\sigma\frac{N_1(t)N_2(t)}{(2\pi)^{3/2}\xi_x\xi_y\xi_z}\exp\left(-\frac{(vt)^2}{2\xi_z^2}\right) \tag{3}$$

where $\xi_x = \sqrt{s_{x,1}^2 + s_{x,2}^2}$ and similarly for ξ_y and ξ_z. We assume that the initial numbers of atoms in each cloud are the same, $N_1(-\infty) = N_2(-\infty) = N_0/2$, so that $N_1(t) = N_2(t) = N(t)/2$. Letting $N_f = N(\infty)$, we integrate Supplementary Equation (3) and find that the scattered fraction $S = 1 - (N_f/N_0)$ is

$$S = \frac{\alpha\sigma}{1 + \alpha\sigma}, \tag{4}$$

with density factor $\alpha = N_0/(4\pi\xi_x\xi_y)$.

In our experiment, the non-zero temperatures of the clouds imply that their momentum distributions are Gaussian and not delta functions, and we should therefore consider the effect of collisions where the two collision partners have velocities $\mathbf{v}_1'$ and $\mathbf{v}_2'$ that are different from the nominal values of $\mathbf{v}_1 = v_1\hat{\mathbf{z}}$ and $\mathbf{v}_2 = -v_2\hat{\mathbf{z}}$. There are four major effects. The first occurs when the collision energy is the same as the nominal energy, but the collision axis is rotated from the z-axis by a small angle θ on the order of $\theta \sim \sqrt{\langle(\mathbf{v}_1 \cdot \hat{\mathbf{y}})^2\rangle}/v$. We are primarily interested in the s-wave scattering halo, and, as s-wave scattering is isotropic, this rotation has no effect on the final pattern. For collision energies where we need to include p-wave scattering ($E/k > 100\ \mu$K), the angle of rotation is on the order of 10 mrad and is undetectable in our images.

The second effect of the Gaussian momentum distributions is that the total momentum of the collision partners may not vanish, so the collision halo may have residual velocity in the laboratory frame. An image of the halo, which captures many realizations of the total momentum, will therefore be elongated along the collision axis. We have not observed this elongation in our absorption images of the scattering halo, which is likely due to a combination of the low ratio of thermal to collision energy and the limited expansion time that we allow the halo.

The third effect of non-zero temperature is that collisions will occur at a range of energies, and that this distribution of collision energies will distort the measured Feshbach resonance parameters. For the resonance studied here, the spread in collision energies is less than the width of the Feshbach resonance in the energy domain, $\Gamma(E)/k$, for energies less than 150 μK given $\delta\mu/k = 160$ μK/G obtained from the coupled-channels model. At higher energies, the spread in collision energies broadens the measured resonance, but this has negligible effect on the measurement of the resonance position at the level of precision obtained in this experiment.

The fourth impact of the momentum spread is that the atom clouds expand in the time between their release from the optical traps and when they collide. The time-to-collision is energy-dependent, as the separation distance at which we turn off the traps is fixed, so the density factor α in Supplementary Equation (4) becomes a function of energy $\alpha(E)$. As we assume that the cross-section is given by Eq. (4), and as $\alpha(E)$ enters Supplementary Equation (4) only as the product $\alpha(E)\sigma$, we use $\alpha(E)$ as a free parameter in our fits to Eq. (5) to match the magnitude of the scattered fraction.

Our experiment further deviates from the simple model in that we observe a secondary, outgoing ^{40}K cloud for collisions near the peak cross-section that is caused by multiple scattering[2,3] of ^{40}K atoms with the ^{87}Rb atoms (see Supplementary Fig. 1).

While this would suggest that we cannot neglect the dynamics of the scattered atoms, we have used a direct-simulation Monte Carlo model of the collider[3] to verify that fitting the simple model to simulated data allows us to reliably extract the resonance parameters $\delta_{\text{bg}}(E)$, $\Gamma_B(E)$, and $B_{\text{res}}(E)$.

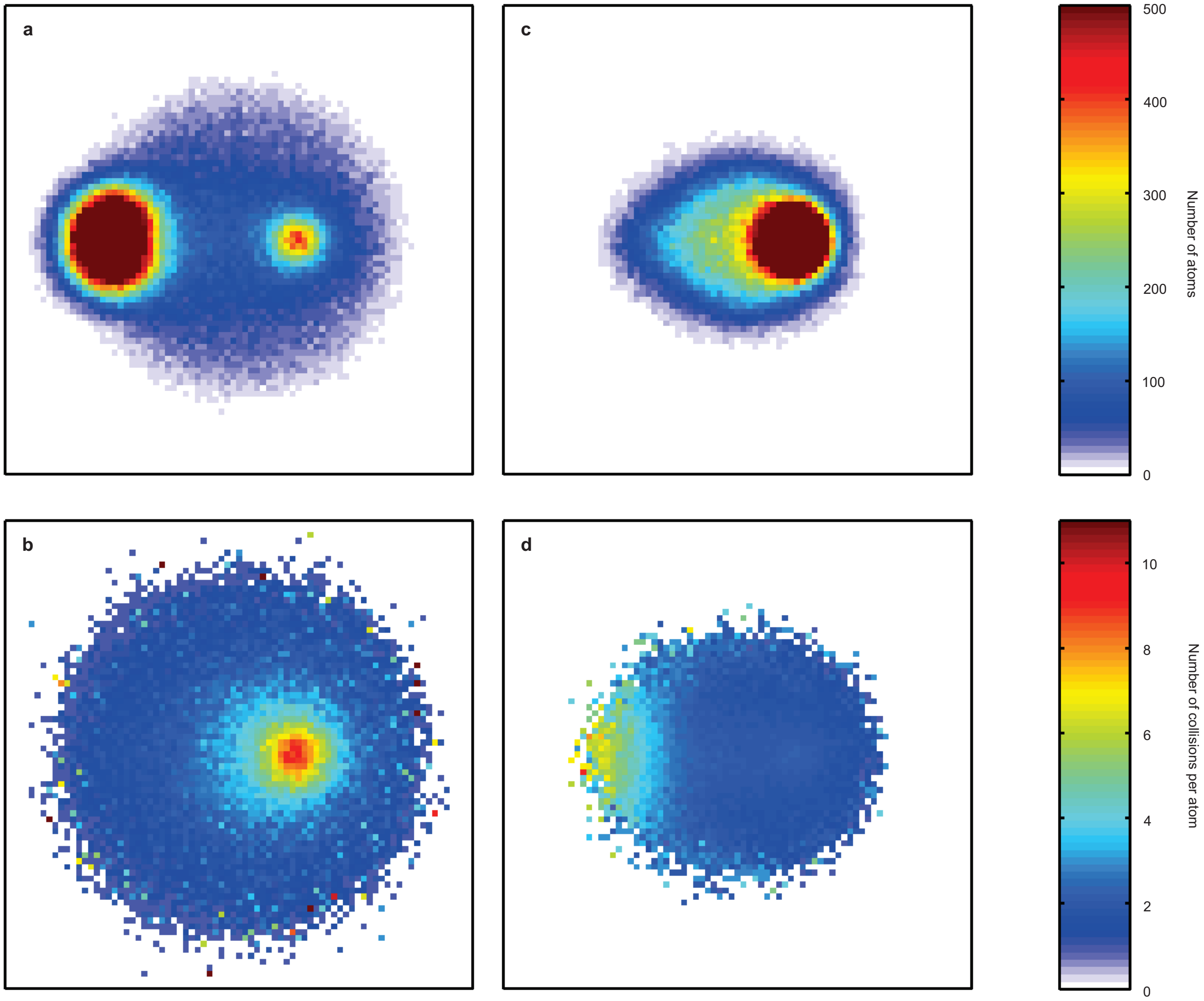

Supplementary Fig. 1 Multiple scattering effects in resonant ^{40}K+^{87}Rb collisions. Simulation of a collision between ^{87}Rb atoms in the $|1,1\rangle$ state and ^{40}K atoms in the $|\frac{9}{2},-\frac{9}{2}\rangle$ state at a collision energy of $E/k = 30\ \mu$K and a magnetic field of $B = 546.6$ G computed using the direct-simulation Monte Carlo method. The 3D spatial distributions are projected onto a 2D plane with a $16 \times 16\ (\mu\text{m})^2$ cell size. The expansion time is 4 ms. **a** Spatial distribution of K atoms after collision. **b** Spatial distribution of Rb atoms after collision computed at the same time-of-flight as for **a**. **c** Spatial distribution of mean number of inter-cloud collisions per cell for K atoms. **d** Spatial distribution of mean number of inter-cloud collisions per cell for Rb atoms. In **a**,**b**, colour indicates number of atoms per cell. In **c**,**d**, colour indicates mean number of inter-cloud collisions per cell. The small secondary cloud in **a** is due to multiple scattering, whereas the same effect leads to a slight elongation of the ^{87}Rb scattering halo in **b**.

SUPPLEMENTARY REFERENCES

[1] Thomas, N. R., Kjærgaard, N., Julienne, P. S. & Wilson, A. C. Imaging of s and d partial-wave interference in quantum scattering of identical bosonic atoms. *Phys. Rev. Lett.* **93**, 173201 (2004).

[2] Kjærgaard, N., Mellish, A. S. & Wilson, A. C. Differential scattering measurements from a collider for ultracold atoms. *New J. Phys.* **6**, 146 (2004).

[3] Thomas, R., Roberts, K. O., Tiesinga, E., Wade, A. C. J., Blakie, P. B., Deb, A. B. & Kjærgaard, N. Multiple scattering dynamics of fermions at an isolated p-wave resonance. *Nat. Commun.* **7**, 12069 (2016).
