# Supplementary material for: Observation of bound state self-interaction in a nano-eV atom collider
Source: Nat Commun. 2018 Nov 20;9:4895. doi: 10.1038/s41467-018-07375-8 (PMC6243998; doi:10.1038/s41467-018-07375-8)
Supplement: Supplementary file 3 — Description of Additional Supplementary Files [file 41467_2018_7375_MOESM3_ESM.pdf]

Description of Additional Supplementary Files

File Name: Supplementary Movie 1
Description: Scattering from colliding clouds of potassium and rubidium. The movie sequence presents experimental data (absorption images acquired at different time delays) that illustrates the operation of the heteronuclear optical collider. The first frame of the sequence corresponds to Fig. 2a of the manuscript where separated ultracold clouds of 40K and 87Rb (prepared in internal quantum states | 9/2,-9/2> and |1,1>, respectively) are held in crossed optical dipole traps. Using steerable optical tweezers the clouds are accelerated to collide in free space under the influence of a homogenous magnetic field that can tune the K+Rb interaction around a Feshbach resonance at 546 G. The movie shows the case of scattering at near the peak cross-section (see Fig. 3a) for particles colliding at an energy of $E/k = 26$ μK (~ 2 neV). The final frame of the sequence corresponds to the situation of Fig. 2e where the clouds have collided and a rubidium scattering halo emerges, surrounded by a halo of potassium atoms. K and Rb atoms are imaged separately using laser light at 767 nm and 780 nm, respectively, as shown in the top and center panels. The bottom panel shows the combined K and Rb images.
